# Substantial Improvements in Facial Morphology through Surgical-Orthodontic Treatment: A Case Report and Literature Review

**DOI:** 10.3390/medicina58081043

**Published:** 2022-08-03

**Authors:** Luminița Ligia Vaida, Bianca Maria Negruțiu, Irina Nicoleta Zetu, Abel Emanuel Moca, Simion Bran

**Affiliations:** 1 Department of Dentistry, Faculty of Medicine and Pharmacy, University of Oradea, 10 Piața 1 Decembrie Street, 410073 Oradea, Romania; ligia_vaida@yahoo.com; 2 Department of Orthodontics and Dentofacial Orthopedics, Faculty of Dentistry, “Grigore T. Popa” University of Medicine and Pharmacy, 16 Universității Street, 700115 Iași, Romania; 3 Discipline of Maxillofacial Surgery and Implantology, Faculty of Dentistry, “Iuliu Hațieganu” University of Medicine and Pharmacy, 8 Victor Babeș Street, 400012 Cluj-Napoca, Romania; dr_brans@yahoo.com

**Keywords:** orthodontic treatment, orthognathic surgery, facial morphology, case report

## Abstract

*Background and Objectives*: The long face type is associated with excessive vertical facial growth and most often with anterior open bite. In many cases of anterior open bite of high severity associated with bimaxillary dento-alveolar protrusion, lips are unable to form an adequate seal at rest. This leads to many issues, including facial dysmorphism. The aim of this study was to describe the case of a 15 year old girl who addressed the orthodontist in November 2015, having complaints related to the skeletal and dental open bite. *Case Description*: A 15.7 year old patient required a consultation with the orthodontic service for impaired dento-facial aesthetics at rest, smile and speech due to an exaggerated superior protrusion of the upper frontal teeth, labial incompetence with excessive gingival exposure at rest and smile associated with upper and lower anterior teeth crowding. The orthodontic diagnostic consisted of skeletal open bite with a hyperleptoprosop morphological facial type, high degree of hyperdivergence, bimaxillary dento-alveolar protrusion, 7 mm skeletal open-bite, 3 mm vertical inocclusion of the anterior teeth, skeletal class II relationships, bilateral half cusp class II molar and canine relationships, labial incompetence, highly increased interlabial gap, facial asymmetry, excessive gingival exposure of 7 mm at smiling and bimaxillary anterior crowding. Because the patient initially refused orthognathic surgery, prior to starting the orthodontic treatment, the patient was recommended to receive a bilateral extraction of the first upper premolars. Key objectives of pre-surgical orthodontic treatment were to achieve a retroclined position of the upper incisors under their normal inclination for the planned upward maxillary rotation, to maintain slightly lower incisor proclination. The orthognathic surgery consisted of Le Fort I impaction osteotomy with 8 mm anterior impactation, bilateral sagittal split osteotomy, and mandibular repositioning using occlusal splint. *Conclusions*: At the end of the orthodontic-surgical treatment, the patient presented significant improvement in dento-facial aesthetics, and optimal skeletal, muscular and dental balance.

## 1. Introduction

Facial appearance has a particularly important contribution to the identity of a person. The study of human facial morphology is an extremely complex area considering the multitude of facial structures, which are represented by soft tissues and hard tissues. These anatomic features can present many quantitative and qualitative variations. Facial development patterns and facial features, including changes in the proportions of the length and width of some facial characteristics, are significantly influenced by environmental factors such as culinary habits and their involvement in the functional capacity and the size of masticatory muscles, swallowing patterns, breathing patterns, and oral habits [1].

There are three basic types of facial morphology, which are as follows: long, normal, and short. The long face type is associated with excessive vertical facial growth and most often with anterior open bite (increased lower anterior facial height, increased gonial angle, increased maxillary/mandibular plane angle) [2,3].

Many authors have investigated in their studies the various correlations between facial morphology and the size of dental arches (in terms of length and width), the shape of dental arches, thickness and activity of masticatory muscles, bite forces, position of the tongue, etc. [4,5,6,7,8,9,10]. It is generally accepted that a stronger, thicker and hyper-functional masticatory muscle can influence bone morphology, thus increasing the transversal dimension of the dento-alveolar arches and leading to a short face morphological type [3,4,5,6]. On the other hand, a thinner and hypo-functional masticatory muscle, particularly with regard to the masseter muscle, is associated with a reduced transversal dimension of dento-alveolar arches and a long face morphological type [7,8,10].

The relationship between malocclusions and facial morphology has been extensively studied by orthodontists since the early 20th century [3]. In a study that analysed the correlation between malocclusion prevalence and facial typology, the authors found that the prevailing facial type in a sample of children from Romania was mesoprosopic (66.24%), followed by leptoprosopic (26.92%) and by euryprosopic (6.84%) [11].

It is well-known that sagittal and vertical alterations in the intermaxillary incisal angle and skeletal intermaxillary malrelationships influence facial morphology especially in patients with class II malocclusions and vertical discrepancies [12,13]. Another study that evaluated the correlation between vertical facial morphology and over-jet in untreated Class II subjects showed a positive association between the overjet and the tendency toward a hyperdivergent pattern. In this study, subjects with an increased overjet (more than 6 mm) had an anterior inclination of the maxilla and vertical facial pattern whereas subjects with normal overjet showed a posterior inclination of the maxilla and horizontal facial pattern [14]. In dental arches correlated with different vertical facial patterns, Sharma A. showed a change in the shape of the upper arch with a proportionally greater narrowing in long face patients, regardless of malocclusion [15].

Some studies reported that a dominant feature of Class II malocclusion is the protrusion of the maxilla [16,17], while other studies suggested a normal maxillary position in patients with Class II malocclusion [18,19].

Siriwat and Jarabak emphasised that a neutral growth pattern was dominant in Class II/I malocclusion, and a hypodivergent pattern was dominant in Class II/2 malocclusion whereas Al-Sairafi found a hyperdivergent pattern in Class II division I subjects [20,21].

In many cases of anterior open bite of high severity associated with bimaxillary dento-alveolar protrusion, lips are unable to form an adequate seal at rest (labial incompetence). Skeletal intermaxillary discrepancies and dental malocclusions are the main factors that influence facial imbalance [22].

Improving facial dysmorphisms in patients with malocclusions may be achieved through an interdisciplinary orthodontic–orthognathic surgical approach of dental, skeletal, and soft tissues features.

The aim of this study was to describe the case of a 15.7-year-old girl who attended the orthodontist’s in November, 2015, with the chief complaints of protrusive upper incisors, lack of lip contact at rest and a large amount of gingival exposure in smile and speech.

## 2. Case Description

### 2.1. Patient Data

A 15.7-year-old patient required a consultation with the orthodontic service for impaired dento-facial aesthetics at rest, smile and speech due to an exaggerated superior protrusion of the upper frontal teeth, labial incompetence with excessive gingival exposure at rest and in smile associated with upper and lower anterior teeth crowding. The patient claimed no previous orthodontic treatment, medical and dental history was not relevant, and the bilateral clinical examination of the temporomandibular joint revealed no abnormal changes with an appropriate range of movements. The patient was included in the orthodontic treatment with her mother’s informed consent. By the time this article was written, the patient was over 18 years old and provided her consent for the patient’s images and other clinical information to be reported in this article. We informed the patient that her name and initials would not be published and we have tried to ensure her identity is concealed, but we cannot guarantee anonymity. The study was conducted in accordance with the World Medical Association (WMA) Declaration of Helsinki—Ethical Principles for Medical Research Involving Human Subjects.

### 2.2. Results of Clinical and Paraclinical Examinations

In order to establish the orthodontic diagnosis, we completed the following steps: anamnesis, general summary clinical examination, loco-regional examination, facial examination, temporo-mandibular joint examination, smile analysis, the analysis of the dimension of interlabial gap at rest, intra-oral examination, functional examination, examination of the study cast, radiographic examination that consisted of orthopantomographic analysis and cephalometric analysis.

**Figure 1:**
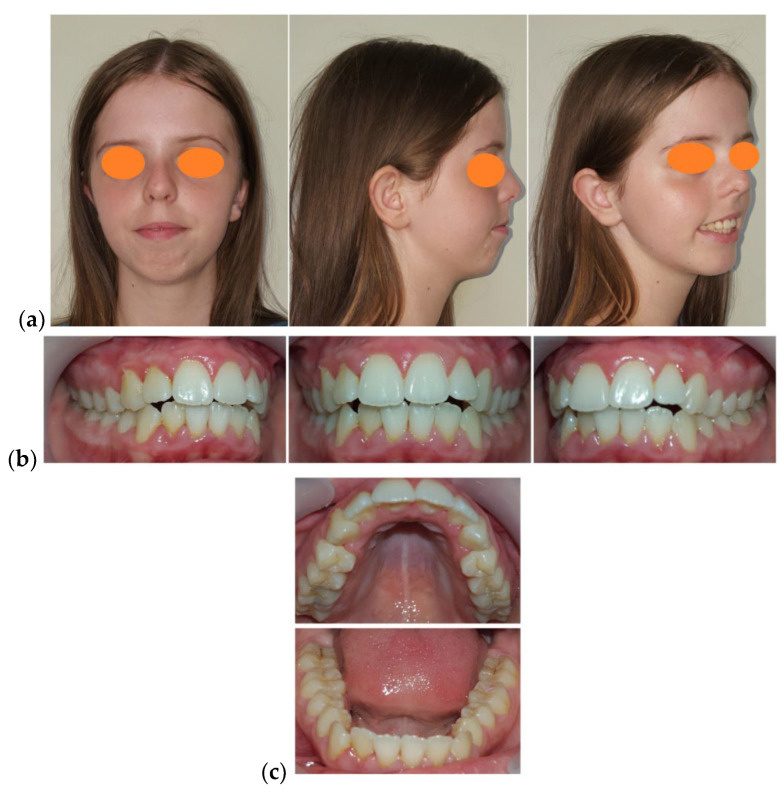
Initial photographic examination: (**a**) Facial aspect: frontal view, profile, ¾ profile on smiling. (**b**) Intra-oral aspect: lateral right, frontal, and lateral left. (**c**) Intra-oral aspect: occlusal view.

The patient’s facial examination revealed a marked leptoprosop morphological type, increased lower anterior face height, asymmetry caused by the deviation of the menton to the left side, narrow nose base, convex facial profile, labial incompetence, unaesthetic hypercontraction of the mentalis muscle causing wrinkling of the chin skin, normal value of the naso-labial angle (>110°), interlabial gap at rest of 14 mm, short upper lip (14.5 mm), V-shaped Cupid’s bow and indentation in middle groove and excessive gingival exposure at rest and at smile (Figure 1a).

The clinical examination of the temporomandibular joint did not reveal any abnormal alterations.

Smile analysis revealed a gingival exposure of 7 mm, vertical maxillary excess, exposure of the lateral teeth from the 1st right premolar to the 1st left premolar, wide buccal corridors, inflamed interdental gingival papillae, the shape and proportion of the teeth were preserved, bimaxillary dento-alveolar protrusion, and deviation of the lower inter-incisor line to the left by 2 mm (Figure 1b). The smile analysis was performed twice, at an interval of 15 min by a single investigator (B.M.N.) and the results were very similar.

Intraoral examination revealed maxillary and mandibular permanent complete dentition, bilateral class II molar and canine, 3 mm over-jet, vertical inocclusion from left canine to right canine, 2 mm deviation of the lower inter-incisor line to the left, Ω” shape of the upper dental-arch (narrowed in the premolars area), protrusion and crowding of the upper anterior teeth, reversed curve of Spee, crowding and protrusion of the lower anterior teeth, and mild gingivitis. Static occlusion examination revealed the presence of a bilateral half cusp distalisation relationship of the first permanent molar and of the canine, sagittal and vertical inocclusion in the anterior teeth, and divergent upper and lower curves of Spee. A dynamic occlusion examination revealed active and passive interferences in propulsion and lateral movements, with reduced possibility of mandibular propulsion (Figure 1b,c).

As for the examination of the dento-maxillary functions, swallowing was evaluated using the Payne technique [23], which showed an atypical function with excessive tongue pressure on the upper and lower anterior teeth, alteration of the physiognomy, poor incision, oral breathing, and labial incompetence.

The cephalometric analysis was performed using a computerised program, Onyx Ceph^3^ (License type-OSL, version-62). This cephalometric analysis revealed a very high Frankfurt-mandibular plane angle, increased IMPA angle, low SNA angle, low SNB angle, increased IF angle, decreased proportion between the lower posterior facial height and the lower anterior facial height, 5 mm retroarchial profile, 2 mm skeletal class II and 3 mm alveolar class II, 7 mm open-bite, and a short upper lip length compared to the results published by Farkas et al. (1994) 21.8 ± 2.2 mm [24], and by Prabu et al. (2012) 23.4 ± 3.42 mm [25] (Table 1). This cephalometric analysis was performed twice by the same author (A.E.M) at an interval of 30 min and the results were averaged.

**Figure 2:**
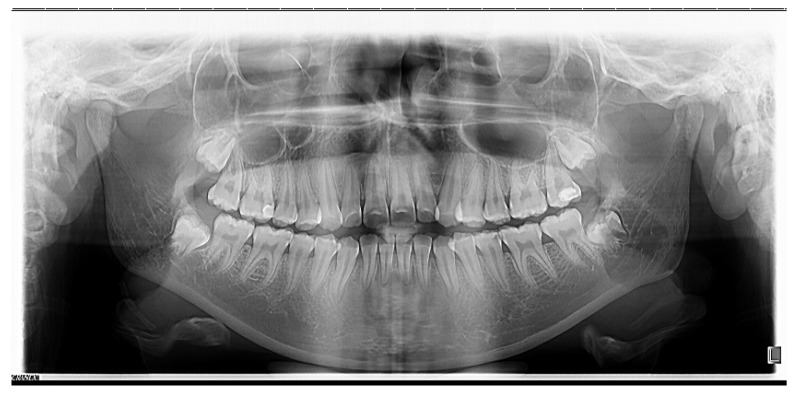
Initial orthopantomography.

The orthopantomographic examination revealed (Figure 2) the presence of all 3rd molars, the mesio-occlusal coronary reconstruction of the first right upper molar and the disto-occlusal of the left upper molar.

**Figure 3:**
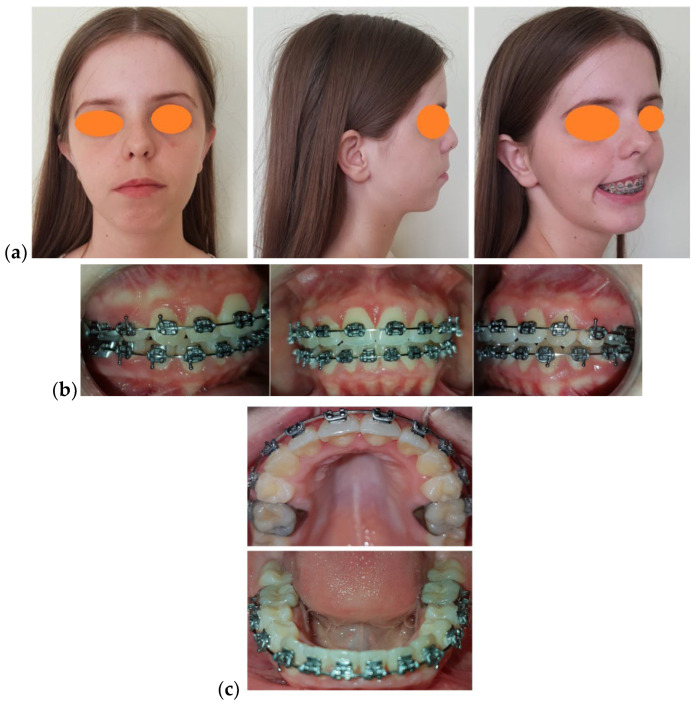
Pre-surgical photographic examination: (**a**) Facial aspect: frontal view, profile, ¾ profile on smiling; (**b**) Intra-oral aspect: lateral right, frontal, and lateral left; (**c**) Intra-oral aspect: occlusal view.

To evaluate the morphological facial type, in addition to the cephalometric parameters we determined the facial morphological index (FMI) using the direct anthropometric method with a millimetric digital sliding caliper. The mean value reported by Da Silva et al. [26] for Caucasians was 81.9. The patient in this study had a FMI (N-Gn/Zy-Zy) of 111.66. (Figure 3, Table 1). The determination of the FMI was performed twice by the same investigator (I.N.Z.) at an interval of 15 min and data were expressed as average.

### 2.3. Morphological Orthodontic Diagnosis

The morphological orthodontic diagnosis included the following:

- Skeletal open bite in a 15.7-year-old patient with hyperleptoprosop morphological facial type (N-Gn/Zy-Zy = 111.66), high degree of hyperdivergence (FMA = 39.22°), bimaxillary dento-alveolar protrusion (IF = 120.85°, IMPA = 98.03°), 7 mm skeletal open-bite, and 3 mm vertical inocclusion of the anterior teeth;
- Skeletal class II relationships (SNA = 78.32°, SNB = 73.98°), bilateral half cusp class II molar and canine relationships;
- Labial incompetence, short upper lip, highly increased interlabial gap;
- Facial asymmetry;
- Excessive gingival exposure of 7 mm in smile;
- Bimaxillary anterior crowding.

### 2.4. Therapeutic Objectives

Based on the results of the clinical and paraclinical examinations presented above, the main therapeutic objective was to achieve a dento-facial balance, to correct the dento-facial dysmorphology in order to obtain a mesoprosop facial morphology type, sufficient coverage at the incisors, optimal gingival exposure as well as a stable and functional occlusal relationship. Achieving this goal was possible only through an orthodontic-surgical interdisciplinary approach.

Unfortunately, at the beginning of the treatment, the patient and her parents refused to consider orthognathic surgery so we proposed using miniscrews or miniplates for treating the gummy smile and improving the vertical dimension of the face. This last treatment option was also refused due to financial reasons and in the end the patient chose only to treat the dental problems. When the orthodontic treatment was finished, the patient, a flute singer, found that when performing it was still quite difficult to maintain lips in contact, so she decided to undergo orthognathic surgery.

Other treatment objectives were as follows: functional re-education of breathing and swallowing, reduction of lower anterior facial height by maxillo–mandibular complex rotation and reduced steepness of occlusal and mandibular planes, correction of skeletal class II discrepancy and facial asymmetry, mentalis muscle relaxation, correction of upper and lower anterior teeth protrusion, alignment of maxillary and mandibular teeth, prevention of flaring of lower incisors, correction of sagittal and vertical inocclusion, and correction of central line deviation.

### 2.5. Initial Combined Treatment Plan

Crowding and severe upper protrusion were the major factors taken into account when we decided to extract the two first premolars as part of the orthodontic treatment for the upper arch (initial discrepancy = −7.5) [27]. The mild dental crowding and the acceptable value of the IMPA angle oriented us towards stripping in the lower arch and finishing orthodontic treatment in canine class I and therapeutic molar class II (initial discrepancy = −3.4).

Prior to starting the orthodontic treatment, the patient was recommended the bilateral extraction of the first upper premolars. Additionally, the odontectomy of all 3rd molars was indicated. For these procedures, it was necessary to obtain the informed consent of both the patient and her mother. The next step consisted of bonding a bimaxillary, non-physiognomic fixed orthodontic appliance. Treatment sequence and treatment biomechanics followed the principles of the Straight-Wire technique as well as the treatment methods that provided a medium anchorage in the upper dental arch.

An important phase in the orthodontic treatment consisted of retracting the upper canines in order to obtain a correct occlusal relationship partially using the space resulting from the extraction of the upper first premolars. The residual space was closed by the orthodontic mesialisation of the upper molars. This phase was followed by “en masse” retraction of the upper incisors. To correct the lower incisors protrusion and achieve the alignment of the lower dental arch we performed IPR using a 0.5 mm/interdental contact point. During pre-surgical orthodontics, we periodically checked the dental arches coordination using the study casts. Key objectives of pre-surgical orthodontic treatment were as follows: to achieve a retroclined position of the upper incisors under their normal inclination for the planned upward maxillary rotation, to maintain slightly lower incisor proclination since the upward surgical rotation of the mandible retroclines them to the true vertical plane, to level upper and lower arches, and to achieve arch coordination. The last arch wires used for the final phase of the pre-surgical orthodontic preparation were made of stainless steel and sized 0.019 × 0.025” (Figure 3).

After approximately 24 months of pre-surgical orthodontic treatment, the patient underwent orthognathic surgery. Prior to orthognathic surgery, occlusal splints were made using an orthognathic articulator. Due to the fact that the patient was already in stage CS6 according to Baccetti et al. [28,29] since the beginning of the treatment at the age of 15.7 and that her flute singing was still altered, it was decided that surgery would take place around the age of 17.6 years, under general anaesthesia and nasal intubation.

### 2.6. Intraoperative Considerations

**Figure 4:**
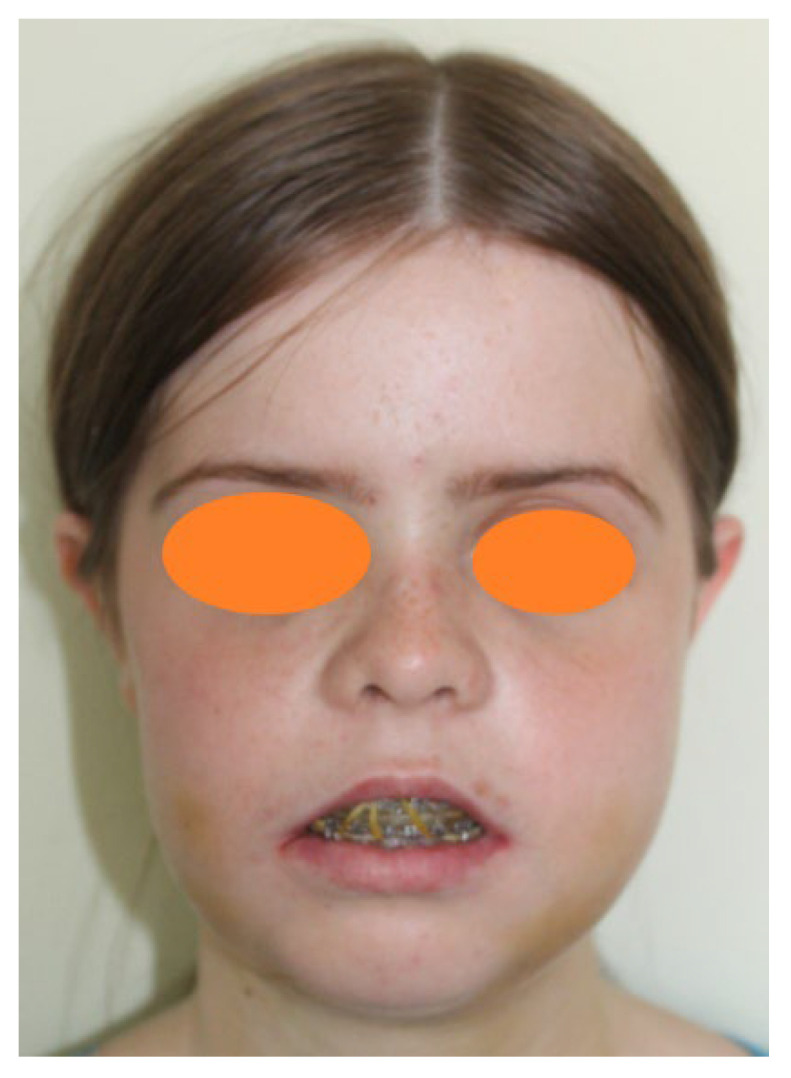
Immediate post-surgical photographic examination. Facial aspect: frontal view with occlusal splint.

The orthognathic surgery consisted of Le Fort I impaction osteotomy with 8 mm anterior impactation, bilateral sagittal split osteotomy (BSSO), ‘inverted L’ osteotomy producing an anti-clockwise rotation of the mandible, mandibular repositioning using occlusal splint (Figure 4), osteosynthesis of the jaw with 4 pads/miniplates and 12 screws, left mandibular osteosynthesis with a pad, 4 bolts and 2 transcortical screws, right mandibular osteosynthesis with 2 pads and 8 screws, and intraoral suture.

The occlusal splint was removed six weeks after orthognathic surgery. The post-surgical orthodontic treatment phase for occlusal refinement lasted 4 months.

### 2.7. Treatment Outcome

Following the orthognathic surgery, all cephalometric parameters improved, reaching normal values (Table 1).

**Figure 5:**
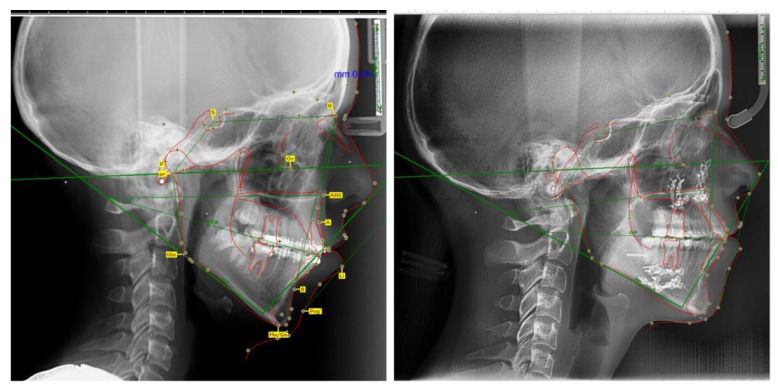
Cephalometric tracing using Onixceph software before (**left**) and after orthognathic surgery (**right**).

The orthognathic surgery resulted in the correction of the patient’s facial morphology by obtaining a mesoprosop facial type. The lower anterior facial height reduced significantly, with a facial index value within the normal range (Table 1). Chin position, interlabial gap and lip competence were also greatly improved (Figure 5).

**Figure 6:**
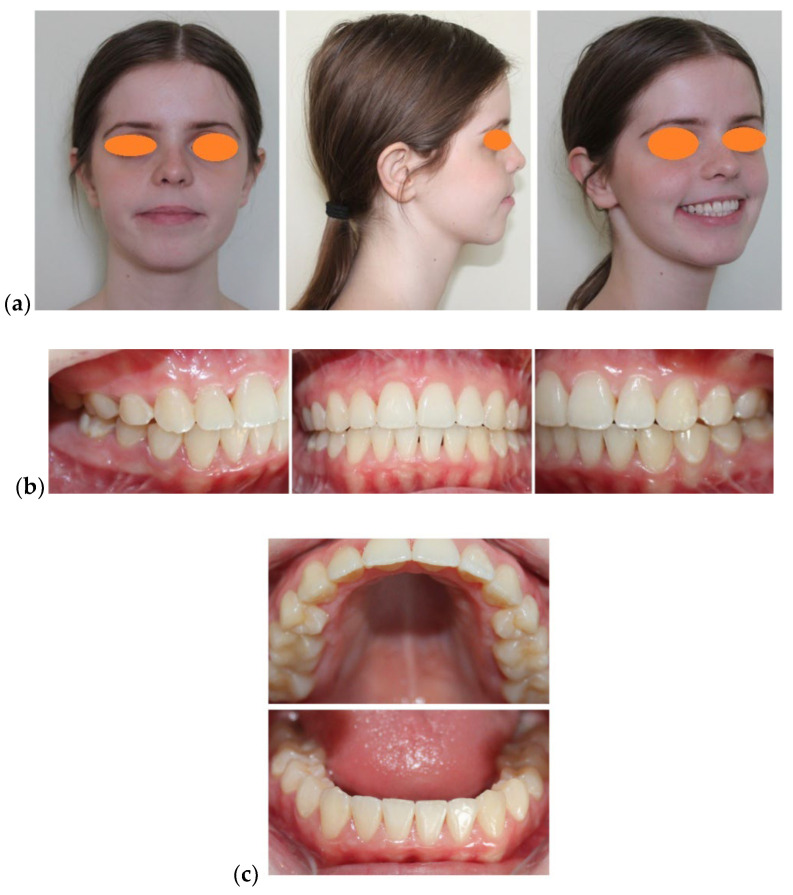
After debonding photographic examination: (**a**) Facial aspect: frontal view, profile, ¾ profile on smiling; (**b**) Intra-oral aspect: lateral right, frontal and lateral left; (**c**) Intra-oral aspect: occlusal view.

The patient’s facial appearance at the end of the orthodontic-surgical treatment was a harmonious one, with a significant improvement of the dento-facial aesthetics, with optimal skeletal, muscular and dental balance (a class I canine occlusal relationship and a stable therapeutic full class II molar occlusal relationship) (Figure 6).

**Figure 7:**
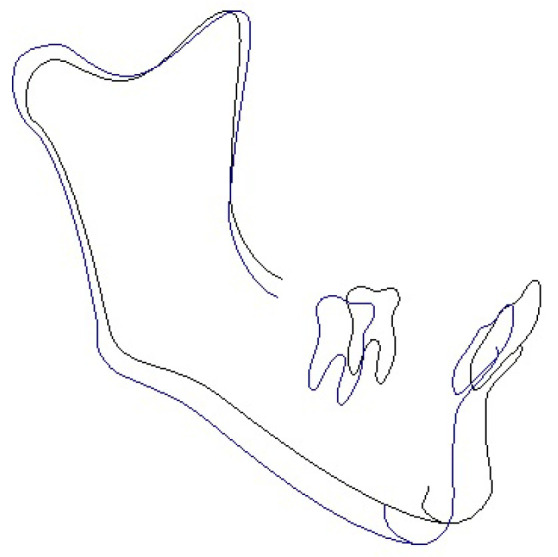

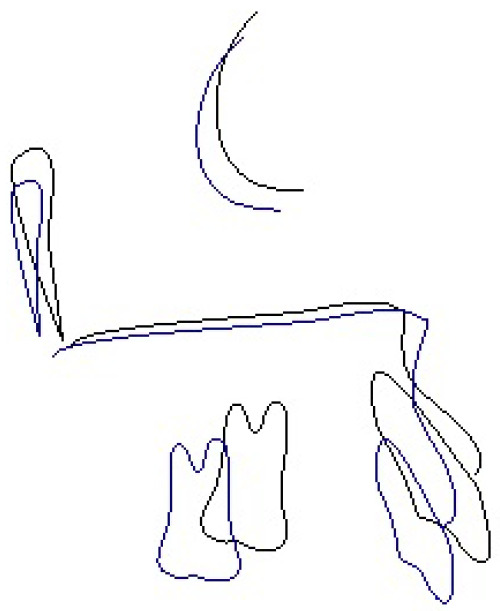
Superimposed tracings of the initial (blue line) and final (black line) cephalometrics.

Superimposed initial and final cephalometric tracings allow us to assess treatment changes (Figure 7).

**Figure 8:**
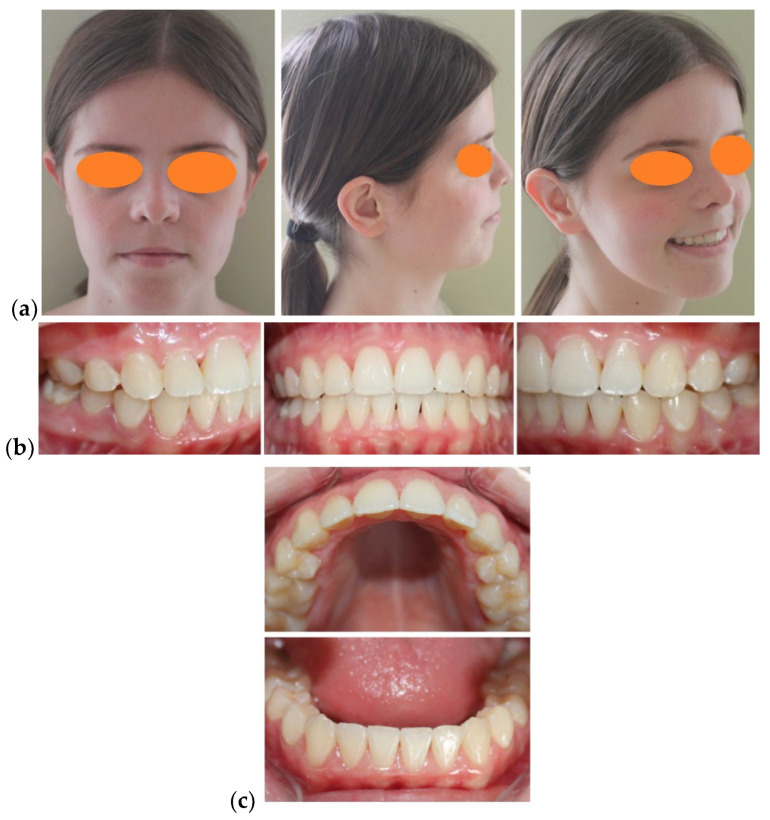
After 24 months from debonding photographic examination: (**a**) Facial aspect: frontal view, profile, ¾ profile on smiling; (**b**) Intra-oral aspect: lateral right, frontal and lateral left; (**c**) Intra-oral aspect: occlusal view.

**Figure 9:**
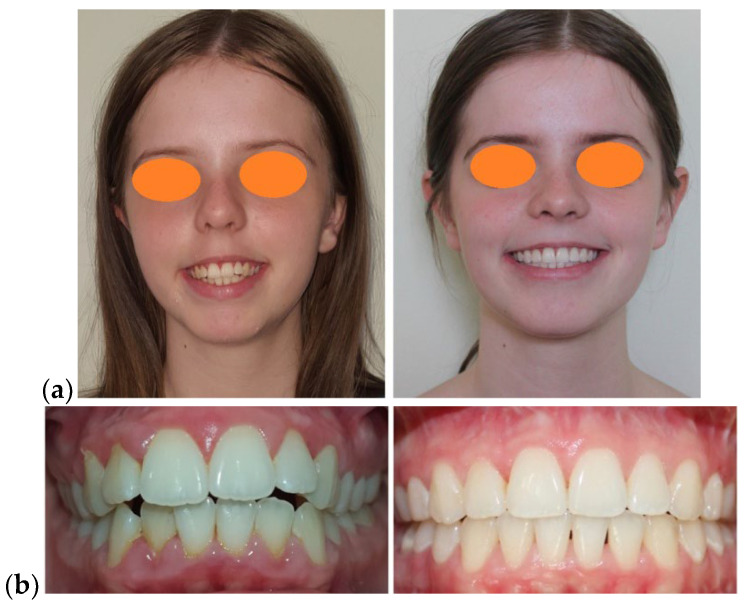
Comparative examination: (**a**) Facial aspect: frontal view smiling; (**b**) Intra-oral aspect: frontal view.

After two years of retention the results were steady (Figure 8 and Figure 9), despite the fact that some authors claim that the more the body changes, the more likely it is for treatment to relapse [30].

## 3. Discussion

The main reasons for the patient’s request for orthodontic treatment were severe protrusion and crowding of the upper anterior teeth, difficulty in keeping her lips in contact and excessive gummy smile. The patient noticed this impossibility of touching her lips during flute classes. Her interest in musical classes further motivated her to achieve the initial proposed goals, increasing adherence and compliance with treatment, thus speeding up the surgery that was performed shortly before the age of 18, mostly because she was already in stage CS6 since the beginning of the treatment (Figure 5).

The patient’s facial dysmorphology was strongly influenced by a short mandibular ramus accompanied by an extreme backward mandibular growth pattern and a long facial type.

One can notice that after the surgical impaction of the maxilla, the upper lip appeared longer and less contracted. The orthognathic surgery led to substantial changes in the facial morphology, the face became mesoprosop; at the same time, the relaxation of the mentalis muscles as well as a more harmonious aspect of the patient’s face were observed.

Recent discoveries in medicine have shown that combined orthodontic–orthognathic surgical treatment can yield extraordinary results in cases that would not be treatable with a unidisciplinary orthodontic approach. The osteotomy of the mandibular ramus requires the same medication as the odontectomy of the third molars, while maxillary osteotomy is better tolerated by patients. However, prior to surgery, a psychological assessment of the patient is required to prepare the patient to easily adapt to the new facial transformations [31]. Thus, clinicians must provide relevant information for every patient’s concerns to make them aware of the risks of general anesthesia and the surgery itself [32] and to provide a realistic description of the possible discomfort following surgery [33]. Our interdisciplinary team paid special attention to the patient’s psychological well-being [34].

Repositioning the maxilla by maxillary osteotomy is an effective method of treating patients with vertical excessive maxillary growth. It is recommended especially in patients with labial incompetence, excessive exposure of the anterior maxillary teeth, gummy smile, increased lower anterior facial height, open bite, retrusive menton and class II or class I malocclusions with bimaxillary dento–alveolar protrusion [35,36].

A LeFort I osteotomy consists of a circum-vestibular incision for vestibular osteotomy and tunnelling for palatal osteotomy, and is recommended when vertical repositioning or vertical and sagittal combined repositioning of the maxilla is desired [37].

From a clinical point of view, the overall improvement of facial appearance, predictability and stability of the results make this procedure a very versatile and effective one when relying on good planning, proper execution and particular attention to detail. The maxillary impactation is one of the most stable orthognathic surgical procedures [35]. On the other hand, the vast majority of patients receiving orthodontic treatment should continue this treatment with the retention phase. The patient in our study benefited from a vacuum-formed retainer for the upper arch and a fixed retainer for the lower arch [29].

The overall treatment duration in our study was 29 months, longer than usual because of the orthodontic extractions of the upper first premolars. The duration of pre-surgical treatment is on average 10 months longer in patients with orthodontic tooth extraction in comparison to non-extraction cases [38]. Our primary goal as orthodontists is to perform a non-extraction orthodontic treatment, but complete correction of the protrusion to this patient, necessary for pre-surgical preparation, would not have been possible without orthodontic extraction. Since the orthognathic surgery led to proper occlusal relationships, the post-surgical orthodontic treatment phase for occlusal refinement lasted only 4 months, less than the duration reported by other studies, with an average duration of 5.9 months [39], or even 7.5 months [40].

In general, patients and relatives are reluctant towards orthognathic surgery, for reasons of stress or anxiety, being reluctant even to a treatment plan that involves dental extractions for orthodontic purposes. We also resorted to strict protocols regarding correct oral hygiene during treatment as well as to close collaboration during the contention period [41]. The strong motivation that the patient had regarding the improvement of her dento–facial aesthetics contributed to a good compliance throughout the orthodontic treatment, but also in the contention phase.

Another therapeutic approach recommended in open-bite and long-face cases consists of using miniscrews or miniplates (TADs—temporary anchorage devices) to achieve intrusion of the maxillary posterior teeth, followed by a mandibular autorotation [42,43,44,45]. This method is indicated in cases with moderate and less severe open bite, as was the case of our patient. In addition, the methods based on the use of TADs also aim to stimulate incisor extrusion in order to correct the anterior open bite, definitely contraindicated in the case of our patient who also had a severe gummy smile. Comparative studies on TADs-treated cases and cases treated with orthognathic surgery suggest that the risk of relapse is higher in TADs cases by the partial re-eruption of the intruded teeth [42,46]. According to Scheffler et al., orthognathic surgery to reposition the maxilla superiorly remains the most reliable approach to create significant rotation of the mandible upward and forward, decreasing anterior facial height along with correction of the open bite [42].

Proper diagnosis, treatment planning associated with interdisciplinary discussions on planning all surgical aspects ensure the success of the surgically treated cases. At each of the treatment phases, the collaboration between the orthodontist and the surgeon is essential, as lack of coordination between the two leads to a compromised result [47]. It is known that the orthodontist thinks in millimetres and angles, while the oro-maxillo-facial surgeon thinks in centimetres. Therefore, the regular assessment of the patient throughout the whole treatment process by both disciplinary teams can ensure a favourable evolution. Achieving pre-surgical objectives with rigid arch wires as well as the manufacture of pre-surgical occlusal splints are steps that require increased attention [48].

## 4. Conclusions

When diagnosing a malocclusion associated with a severe skeletal discrepancy that cannot be alleviated by growth, the orthodontic–orthognathic surgical treatment provides a better aesthetical, functional, and stable result. For this patient, the orthodontic-surgical treatment proved to be the appropriate one, with considerable improvements of the facial morphology, but also at the dental and occlusal level, leading to increased facial attractiveness and, implicitly, to increased self-esteem. Clinically, this study emphasised the fact that surgery must always be considered for a proper treatment result. Even though this patient was initially compensated and treated as an extraction case, due to orthognathic surgery refusal, after finally accepting surgery, a positive and well-balanced outcome was still achievable. Patients must be properly informed and reminded of all possible treatment options.

## Figures and Tables

**Table 1:** Pre and post treatment cephalometric measurements.

Parameters	Normal Values	Pre- Treatment	Before Orthognatic Surgery	After Orthognatic Surgery	Differences
FMA	25 ± 3°	39.22°	36.13°	28.23°	10.99°
IMPA	88 ± 3°	98.03°	93.22°	91.14°	6.89°
SNA	82 ± 2°	78.32°	78.17°	80.35°	−2.03°
SNB	80 ± 2°	73.98°	74.12°	78.22°	−4.24°
IF	107° ± 5°	120.85°	107.32°	111.80°	9.05°
HFP/HFA	0.69	0.52	0.54	0.64	0.12
Upper lip length (mm)	23.4 ± 3.42	14.5	14.7	16.8	2.3
FMI	81.9	111.6	110.2	84.3	27.3
U1-SN	103 ± 7°	115.12°	104.78°	109.50°	5.62°

## Data Availability

Not applicable.
